# ﻿Current knowledge on the diversity of Eumolpinae (Coleoptera, Chrysomelidae) in New Caledonia

**DOI:** 10.3897/zookeys.1177.101293

**Published:** 2023-08-30

**Authors:** Leonardo Platania, Jesús Gómez-Zurita

**Affiliations:** 1 Institut Botànic de Barcelona (CSIC-Ajuntament de Barcelona), 08038 Barcelona, Spain Institut Botànic de Barcelona (CSIC- Ajuntament de Barcelona) Barcelona Spain; 2 Universitat Pompeu Fabra, 08003 Barcelona, Spain Universitat Pompeu Fabra Barcelona Spain

**Keywords:** Catalogue, conservation, leaf beetles, Linnean shortfall, South Pacific, synonymies, taxonomy

## Abstract

The Eumolpinae leaf beetles of New Caledonia are very diverse, but our knowledge about their diversity is still incomplete. Following a renewed interest in the group in the last two decades, there has been an exponential increase in the number of species described, with species descriptions and taxonomic reassessment ongoing. In this work, the catalogue of New Caledonian Eumolpinae is updated, incorporating all these recent changes, and also indicating the collection where type specimens are currently available. The updated catalogue includes 120 species in 13 genera, and more additions and taxonomic changes, including new combinations, are expected in forthcoming years. Here two new synonymies are reported, namely *Dumbeastriata* Jolivet, Verma & Mille, 2007 = *Taophilacancellata* Samuelson, 2010, **syn. nov.**; and *Dematochromatheryi* Jolivet, Verma & Mille, 2010 = *Dematochromapoyensis* Jolivet, Verma & Mille, 2010, **syn. nov.** Moreover, two species still retaining their original adscription to the genus *Colaspis* Fabricius, 1801, are treated as incertae sedis. This catalogue represents a useful tool for future taxonomic studies of New Caledonian Chrysomelidae and can assist biodiversity surveys and conservation studies within the archipelago.

## ﻿Introduction

**Figure 1.**
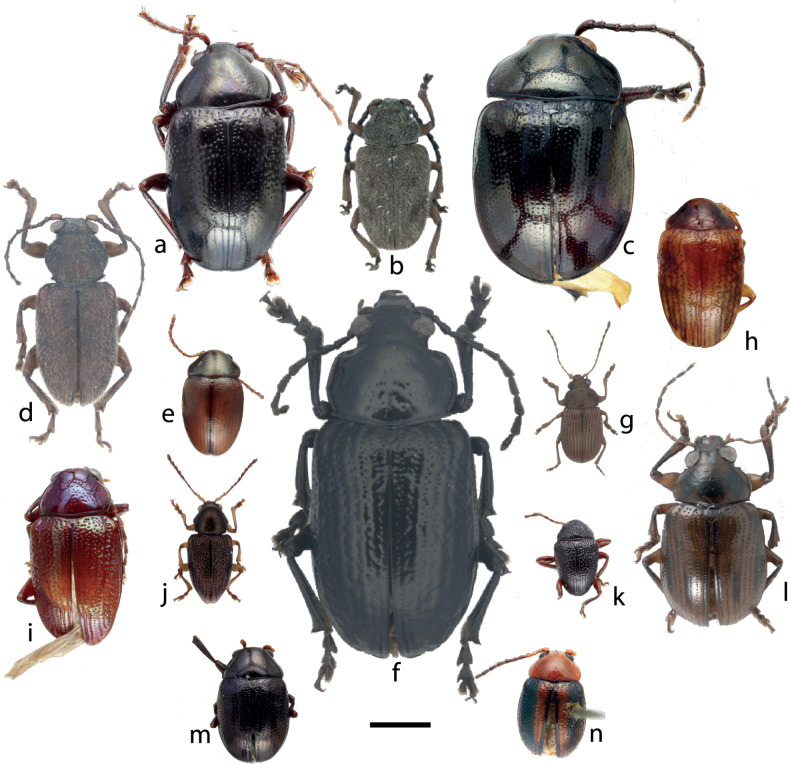
Dorsal views of the holotypes of Eumolpinae of New Caledonia **a***Cazeresiamontana* Jolivet, Verma & Mille, 2005 **b***Tricholapitaolympica* (Platania & Gómez-Zurita, 2020) **c***Colaspoidesfontis* Jolivet, Verma & Mille, 2008 **d***Dematotrichusvillosus* Gómez-Zurita, 2022 **e***Montrouzierellabrinoni* Jolivet, Verma & Mille, 2007 **f***Thasyclesmagnus* Gómez-Zurita, 2022 **g***Kumatoeidesmegale* Gómez-Zurita, 2018 **h***Dumbeamontana* Jolivet, Verma & Mille, 2011 **i***Dematochromatheryi* Jolivet, Verma & Mille, 2010 **j***Taophiladraco* Platania & Gómez-Zurita, 2022 **k***Acronymolpusbertiae* (Jolivet, Verma & Mille, 2007) **l***Rhyparidafoaensis* (Jolivet, Verma & Mille, 2007) **m***Samuelsoniamelas* Jolivet, Verma & Mille, 2007 **n***Colaspissolani* Perroud & Montrouzier, 1864. Scale bar: 2.00 mm.

Within the Chrysomelidae, a highly diverse insect family accounting some 40,000 species (Leschen and Beutel 2014), Eumolpinae is an important subfamily representing approximately 7,000 species and 500 genera, mainly distributed in tropical areas (Jolivet and Verma 2010; Jolivet et al. 2014). Large part of the diversity of Eumolpinae is still unknown (Jolivet and Verma 2010) and their supraspecific systematics is unsatisfactory (Gómez-Zurita et al. 2005; Jolivet et al. 2014, Reid 2017). All issues related to the so-called Linnaean shortfall are relevant for this group, as the limited taxonomic knowledge makes it difficult to advance in many other fields (Lomolino 2004). An important taxonomic gap affects the group across the tropics, including South Pacific islands, where the last relatively comprehensive works on Eumolpinae had been published ~ 50 years ago and were restricted to the archipelagos of Fiji and Samoa, and partially for New Zealand (Gressitt 1956; Bryant and Gressitt 1957; Shaw 1957). This insular region is interesting for this group, because it is disharmonious for the distribution of Chrysomelidae, with several subfamilies missing or poorly represented in native faunas, whereas Eumolpinae are disproportionally diverse, particularly in New Caledonia (Jolivet and Verma 2008; Papadopoulou et al. 2013). The Eumolpinae of New Caledonia, briefly illustrated in Fig. 1, belong to two tribes, the Typophorini, represented by a single species, *Rhyparidafoaensis* (Jolivet et al. 2007a), probably the result of a recent introduction (Gómez-Zurita 2011a), and the Eumolpini, highly diverse, estimated to have more than 200 species, most of them still to be described, and possibly the result of a large radiation in situ (Gómez-Zurita 2011b; Papadopoulou et al. 2013). Apart from the high species richness of Eumolpinae, the geological, geographical, and ecological features of New Caledonia make it particularly interesting to invest on a good knowledge about the diversity and ecology of this group. New Caledonia is an archipelago of relatively small size and with a long history of isolation from the mainland, and it hosts an enormous and nearly entirely endemic diversity across several groups of organisms, having been recognised as a biodiversity hotspot, central for conservation concerns and for the study of island evolution and biogeography (Myers et al. 2000; Grandcolas 2008).

The past fifteen years have seen an increased interest on the diversity of New Caledonian Eumolpinae. The previous knowledge on these beetles was made available in the early works by Xavier Montrouzier (Montrouzier 1861; Perroud and Montrouzier 1864), Albert Fauvel (Fauvel 1862), and Karl M. Heller (Heller 1916). After the passionate rediscovery of this important group of beetles in the fauna of New Caledonia by Pierre Jolivet, Krishna Verma, and Christian Mille, a real renaissance in the taxonomic research of the subfamily took place. In the first years of the new Century, these entomologists started surveying the diversity of New Caledonian Eumolpinae and described numerous species (Jolivet et al. 2005, 2007a, b, c, 2009a, b, 2010, 2013). Allan Samuelson contributed relevant revisionary studies on the genus *Taophila* Heller, 1916 (Samuelson 2010) and described a new genus, *Acronymolpus* Samuelson, 2015, and Lev Medvedev also described a single species of Eumolpinae from New Caledonia together with several other tropical Eumolpinae (Medvedev 2007). More recently, in the past 12 years, our group took on the task to contribute towards the taxonomic knowledge of New Caledonian Eumolpinae, reassessing the data from previous studies and also describing new genera and species (Gómez-Zurita 2011a, b, 2017a, b, 2018, 2020, 2022; Gómez-Zurita and Cardoso 2014; Platania et al. 2020; Gómez-Zurita et al. 2020; Platania and Gómez-Zurita 2022; Gómez-Zurita and Pàmies-Harder 2022). Finally, Mille and Jolivet (2021) published the illustrated catalogue of New Caledonian Chrysomelidae, including the available knowledge on Eumolpinae, although new species and taxonomic rearrangements affected the group while this catalogue was still in production. Here, we report an updated list of Eumolpinae, including 120 species, keeping track of taxonomic and nomenclatural changes, and proposing two new synonymies, to facilitate access to taxonomic knowledge on the New Caledonian fauna of Eumolpinae. We hope that this can become a useful tool to guide forthcoming work on this group, a fundamental task to tackle the Linnaean shortfall, and to deal with the pressing matter of conservation in New Caledonia and elsewhere.

## ﻿Materials and methods

The catalogue is based on all the published information on New Caledonian Eumolpinae, including data on the confirmed availability of the typical series or types, whereby the institution holding the primary type is highlighted in bold in the list below, and paratypes or other material in regular font (an asterisk denotes lack of information about the primary type, although paratypes may be available). The type species of each genus is underlined.

Acronyms of entomological collections and museums reported in the catalogue:

**AMS**Australian Museum, Sydney;

**BPBM**Pauahi Bishop Museum of Polynesian Ethnology and Natural History, Honolulu;

**CXMNC** Collection Xavier Montrouzier, Institut Agronomique néo-Calédonien, La Foa;

**HNHM**Hungarian Natural History Museum, Budapest;

**JGZC** Jesús Gómez-Zurita Collection, CSIC, Barcelona;

**MNHN**Muséum National d’Histoire Naturelle, Paris;

**MNHW** Museum of Natural History, Wrocław University, Wroclaw;

**NHM**Natural History Museum, London;

**NRM**Swedish Museum of Natural History, Stockholm;

**RBINS**Royal Belgian Institute of Natural Sciences, Bruxelles;

**SMTD** Staatliches Museum für Tierkunde, Dresden;

**ZISP**Zoological Institute of Russian Academy of Sciences.

## ﻿Species catalogue

### ﻿Eumolpini

1. *Acronymolpusbertiae* (Jolivet, Verma & Mille, 2007) (Fig. 1k) — Rev. fr. Entomol. 29: 81. (**MNHN**)

= *Acronymolpusmeteorus* Samuelson, 2015 — ZooKeys 547: 100. (**BPBM**)

= *Acronymolpusturbo* Samuelson, 2015 — ZooKeys 547: 97. (**CXMNC/MNHN**)

2. *Acronymolpusjourdani* (Jolivet, Verma & Mille, 2013) — Nouv. Revue Ent. (N.S.) 29: 145. (MNHN)*

= *Acronymolpusgressitti* Samuelson, 2015 — ZooKeys 547: 99. (**BPBM**)

= *Acronymolpusjoliveti* Samuelson, 2015 — ZooKeys 547: 95. (**BPBM**)

3. *Cazeresiamontana* Jolivet, Verma & Mille, 2005 (Fig. 1a) — Rev. fr. Entomol. 27: 70. (**MNHN**)

4. *Colaspoidescaledonica* Medvedev, 2007 — Euroasian Ent. J. 6(4): 434. (**ZISP**)

5. *Colaspoidesfontis* Jolivet, Verma & Mille, 2008 (Fig. 1c) — Nouv. Revue Ent. (N.S.) 24: 198. (**MNHN**)

6. *Colaspoideskanalensis* (Perroud & Montrouzier, 1864) — Annls. Soc. linn. Lyon 11: 207. (**MNHN**)

7. *Colaspoidessarrameae* Jolivet, Verma & Mille, 2008 — Nouv. Revue Ent. (N.S.) 24: 198. (**MNHN**)

8. *Dematochromaantipodum* (Fauvel, 1862) — Bull. Soc. Linn. Normandie 7: 167. (**MNHN**)

9. *Dematochromaculminicola* (Heller, 1916) — Sarasin and Roux, Nova Caled., Zool. 2: 304. (**SMTD**)

10. *Dematochromadifficilis* (Heller, 1916) — Sarasin and Roux, Nova Caled., Zool. 2: 305. (**SMTD**)

11. *Dematochromadoiana* Jolivet, Verma & Mille, 2007 — Rev. fr. Entomol. 29: 38. (MNHN)*

12. *Dematochromahelleri* Jolivet, Verma & Mille, 2007 — Rev. fr. Entomol. 29: 42. (**MNHN**)

13. *Dematochromahumboldtiana* (Heller, 1916) — Sarasin and Roux, Nova Caled., Zool. 2: 301. (**SMTD**)

14. *Dematochromalepros* (Heller, 1916) — Sarasin and Roux, Nova Caled., Zool. 2: 301. (**SMTD**)

15. *Dematochromamaculifrons* (Heller, 1916) — Sarasin and Roux, Nova Caled., Zool. 2: 302. (**SMTD**)

16. *Dematochromasamuelsoni* Jolivet, Verma & Mille, 2011 — Nouv. Revue Ent. (N.S.) 26: 334. (**MNHN**)

17. *Dematochromasylviae* Jolivet, Verma & Mille, 2010 — Nouv. Revue Ent. (N.S.) 26: 10. (**MNHN**)

18. *Dematochromaterastiomerus* (Heller, 1916) — Sarasin and Roux, Nova Caled., Zool. 2: 303. (**SMTD**)

19. *Dematochromaterminaliae* Jolivet, Verma & Mille, 2010 — Nouv. Revue Ent. (N.S.) 26: 10. (**MNHN**)

20. *Dematochromatheryi* Jolivet, Verma & Mille, 2010 (Fig. 1i) — Nouv. Revue Ent. (N.S.) 26: 12. (**MNHN**)

= *Dematochromapoyensis* Jolivet, Verma & Mille, 2010, syn. nov. — Nouv. Revue Ent. (N.S.) 26: 12 (**MNHN**)

21. *Dematochromathyiana* Jolivet, Verma & Mille, 2008 — Nouv. Revue Ent. (N.S.) 24: 196. (**MNHN**)

22. *Dematotrichuscapillaris* Gómez-Zurita, 2022 — System. Biodivers. 20: 8. (**JGZC**)

23. *Dematotrichuscapillosus* Gómez-Zurita, 2022 — System. Biodivers. 20: 12. (**MNHW**)

24. *Dematotrichuscomans* Gómez-Zurita, 2022 — System. Biodivers. 20: 13. (**MNHW**)

25. *Dematotrichuscomatulus* Gómez-Zurita, 2022 — System. Biodivers. 20: 15. (**JGZC**, MNHW, MNHN)

26. *Dematotrichuscrinitus* Gómez-Zurita, 2022 — System. Biodivers. 20: 16. (**MNHW**, JGZC)

27. *Dematotrichushirsutus* Gómez-Zurita, 2022 — System. Biodivers. 20: 17. (**JGZC**, MNHW, MNHN)

28. *Dematotrichushirtus* Gómez-Zurita, 2022 — System. Biodivers. 20: 18. (**JGZC**, MNHW, MNHN)

29. *Dematotrichushispidus* (Jolivet, Verma & Mille, 2013) — Nouv. Revue Ent. (N.S.) 29: 152. (MNHN)*

30. *Dematotrichushorridus* Gómez-Zurita, 2022 — System. Biodivers. 20: 21. (**MNHW**)

31. *Dematotrichuspilosus* (Jolivet, Verma & Mille, 2007) — Rev. fr. Entomol. 29: 38. (**MNHN**)

32. *Dematotrichuspubescens* Gómez-Zurita, 2022 — System. Biodivers. 20: 23. (**JGZC**, MNHW)

33. *Dematotrichussetosus* Gómez-Zurita, 2022 — System. Biodivers. 20: 24. (**MNHW**)

34. *Dematotrichusvillosus* Gómez-Zurita, 2022 (Fig. 1d) — System. Biodivers. 20: 25. (**MNHW**, JGZC)

35. *Dumbeagigas* Jolivet, Verma & Mille, 2007 — Rev. fr. Entomol. 29: 81. (**MNHN**)

36. *Dumbeamontana* Jolivet, Verma & Mille, 2011 (Fig. 1h) — Nouv. Revue Ent. (N.S.) 26: 337. (**MNHN**)

37. *Dumbeapaulaudi* Jolivet, Verma & Mille, 2007 — Rev. fr. Entomol. 29: 80. (**MNHN**)

38. *Dumbeastriata* Jolivet, Verma & Mille, 2007 — Rev. fr. Entomol. 29: 80. (**MNHN**)

= *Taophilacancellata* Samuelson, 2010, syn. nov. — Zootaxa 2621: p. 49. (**MNHN**, BPBM)

39. *Edusellaflaveola* (Montrouzier, 1861) — Annls. Soc. ent. Fr. 4: 396*.

40. *Kumatoeidesanomala* Gómez-Zurita, 2018 — Zootaxa 4521: 5. (**MNHN**, MNHW)

41. *Kumatoeidesaulacia* Gómez-Zurita, 2018 — Zootaxa 4521: 6. (**HNHM**)

42. *Kumatoeidescostata* (Jolivet, Verma & Mille, 2007) — Rev. fr. Entomol. 29: 88. (**MNHN**)

43. *Kumatoeidesleptalei* Gómez-Zurita, 2018 — Zootaxa 4521: 11. (**MNHN**, MNHW)

44. *Kumatoeidesmegale* Gómez-Zurita, 2018 (Fig. 1g) — Zootaxa 4521: 12. (**MNHN**, MNHW)

45. *Kumatoeidesmetallica* Gómez-Zurita, 2018 — Zootaxa 4521: 16. (**MNHN**, MNHW)

46. *Kumatoeidesmillei* Gómez-Zurita, 2018 — Zootaxa 4521: 17. (**MNHN**, MNHW)

47. *Kumatoeidestarsalis* Gómez-Zurita, 2018 — Zootaxa 4521: 19. (**MNHN**, MNHW, JGZC)

48. *Kumatoeideswanati* Gómez-Zurita, 2018 — Zootaxa 4521: 22. (**MNHN**, MNHW)

49. *Montrouzierellabrinoni* Jolivet, Verma & Mille, 2007 (Fig. 1e) — Rev. fr. Entomol. 29: 89. (**MNHN**)

50. *Montrouzierellaflava* Jolivet, Verma & Mille, 2007 — Rev. fr. Entomol. 29: 89. (**MNHN**)

51. *Montrouzierellametrosiderosi* Jolivet, Verma & Mille, 2011 — Nouv. Revue Ent. (N.S.) 26: 338. (**MNHN**)

52. *Montrouzierellanana* Jolivet, Verma & Mille, 2007 — Rev. fr. Entomol. 29: 87. (**MNHN**)

53. *Montrouzierellasubtuberculata* Jolivet, Verma & Mille, 2010 — Nouv. Revue Ent. (N.S.) 26: 14. (**MNHN**)

54. *Montrouzierellatuberculata* Jolivet, Verma & Mille, 2007 — Rev. fr. Entomol. 29: 88. (**MNHN**)

55. *Samuelsoniabicolor* Jolivet, Verma & Mille, 2007 — Rev. fr. Entomol. 29: 85. (**MNHN**)

56. *Samuelsoniadunali* (Montrouzier, 1861) — Annls. Soc. ent. Fr. 4: 396. (**RBINS**)

57. *Samuelsoniafauveli* Jolivet, Verma & Mille, 2007 — Rev. fr. Entomol. 29: 87. (**MNHN**)

58. *Samuelsoniafusca* Jolivet, Verma & Mille, 2007 — Rev. fr. Entomol. 29: 84. (**MNHN**)

59. *Samuelsoniagomyi* Jolivet, Verma & Mille, 2013 — Nouv. Revue Ent. (N.S.) 29: 147. (MNHN)*

60. *Samuelsoniahistrio* (Perroud & Montrouzier, 1864) — Annls. Soc. linn. Lyon 11: 205. (**MNHN**)

61. *Samuelsonialemerrei* Jolivet, Verma & Mille, 2013 — Nouv. Revue Ent. (N.S.) 29: 148. (MNHN)*

62. *Samuelsoniamayonae* Jolivet, Verma & Mille, 2010 — Nouv. Revue Ent. (N.S.) 26: 14. (**MNHN**)

63. *Samuelsoniamelas* Jolivet, Verma & Mille, 2007 (Fig. 1m) — Rev. fr. Entomol. 29: 83. (**MNHN**)

64. *Samuelsoniaminima* Jolivet, Verma & Mille, 2013 — Nouv. Revue Ent. (N.S.) 29: 150. (MNHN)*

65. *Samuelsonianitida* Jolivet, Verma & Mille, 2013 — Nouv. Revue Ent. (N.S.) 29: 149. (MNHN)*

66. *Samuelsoniapanieensis* Jolivet, Verma & Mille, 2011 — Nouv. Revue Ent. (N.S.) 26: 336. (**MNHN**)

67. *Samuelsoniapardalis* Jolivet, Verma & Mille, 2007 — Rev. fr. Entomol. 29: 86. (**MNHN**)

68. *Samuelsoniapilosa* Jolivet, Verma & Mille, 2007 — Rev. fr. Entomol. 29: 85. (**MNHN**)

69. *Samuelsoniapygmaea* Jolivet, Verma & Mille, 2010 — Nouv. Revue Ent. (N.S.) 26: 15. (**MNHN**)

70. *Samuelsoniarubiacearum* (Perroud & Montrouzier, 1864) — Annls. Soc. linn. Lyon 11: 203. (**MNHN**)

71. *Samuelsoniarugosa* Jolivet, Verma & Mille, 2013 — Nouv. Revue Ent. (N.S.) 29: 150. (MNHN)*

72. *Samuelsoniaturgida* Jolivet, Verma & Mille, 2007 — Rev. fr. Entomol. 29: 86. (**MNHN**)

73. *Samuelsoniaviridescens* Jolivet, Verma & Mille, 2013 — Nouv. Revue Ent. (N.S.) 29: 151. (MNHN)*

74. Taophila (Jolivetiana) mantillerii Jolivet, Verma & Mille, 2007 — Rev. fr. Entomol. 29: 44. (MNHN)*

75. Taophila (Taophila) bituberculata Platania & Gómez-Zurita, 2022 — Insect Syst. Evol. 53: 13. (**JGZC**)

76. Taophila (Taophila) carinata Platania & Gómez-Zurita, 2022 — Insect Syst. Evol. 53: 17. (**MNHN**, MNHW, JGZC)

77. Taophila (Taophila) corvi Samuelson, 2010 — Zootaxa 2621: 51. (**BPBM**)

78. Taophila (Taophila) dapportoi Platania & Gómez-Zurita, 2022 — Insect Syst. Evol. 53: 25. (**MNHN**, MNHW)

79. Taophila (Taophila) davincii Platania & Gómez-Zurita, 2022 — Insect Syst. Evol. 53: 29. (**MNHN**, MNHW, JGZC)

80. Taophila (Taophila) deimos Samuelson, 2010 — Zootaxa 2621: 53. (**BPBM**)

81. Taophila (Taophila) draco Platania & Gómez-Zurita, 2022 (Fig. 1j) — Insect Syst. Evol. 53: 33. (**MNHN**, MNHW)

82. Taophila (Taophila) goa Platania & Gómez-Zurita, 2022 — Insect Syst. Evol. 53: 38. (**MNHN**, MNHW, JGZC)

83. Taophila (Taophila) hackae Platania & Gómez-Zurita, 2022 — Insect Syst. Evol. 53: 42. (**JGZC**)

84. Taophila (Taophila) hydrae Samuelson, 2010 — Zootaxa 2621: 53. (**BPBM**)

85. Taophila (Taophila) joliveti Samuelson, 2010 — Zootaxa 2621: 54. (**BPBM**)

86. Taophila (Taophila) millei Samuelson, 2010 — Zootaxa 2621: 58. (**BPBM**)

87. Taophila (Taophila) nigrans Jolivet, Verma & Mille, 2007 — Rev. fr. Entomol. 29: 44. (**MNHN**)

88. Taophila (Taophila) sagittarii Samuelson, 2010 — Zootaxa 2621: 58. (**BPBM**)

89. Taophila (Taophila) samuelsoni Platania & Gómez-Zurita, 2022 — Insect Syst. Evol. 53: 50. (**MNHN**, MNHW)

90. Taophila (Taophila) scorpii Samuelson, 2010 — Zootaxa 2621: 59. (**BPBM**, MNHN)

91. Taophila (Taophila) sideralis Platania & Gómez-Zurita, 2022 — Insect Syst. Evol. 53: 53. (**MNHN**, MNHW, JGZC)

92. Taophila (Taophila) subsericea Heller, 1916 — Sarasin and Roux, Nova Caled., Zool., 2: 306. (**SMTD**)

= *Stethotesmandjeliae* Jolivet, Verma & Mille, 2010 — Rev. fr. Entomol. 32: 143. (**MNHN**)

93. Taophila (Taophila) taaluny Platania & Gómez-Zurita, 2022 — Insect Syst. Evol. 53: 59. (**MNHN**)

94. Taophila (Taophila) wanati Platania & Gómez-Zurita, 2022 — Insect Syst. Evol. 53: 61. (**MNHN**, MNHW, JGZC)

95. *Thasyclescastaneus* Gómez-Zurita, 2022 — Zool. Anz. 297: 24. (**MNHW**)

96. *Thasyclescompactus* Gómez-Zurita, 2022 — Zool. Anz. 297: 25. (**JGZC**, MNHW)

97. *Thasyclescordiformis* Chapuis, 1874 — Hist. nat. Ins., Gen. Col. X: p. 255. (**RBINS**)

98. *Thasyclesfuscus* (Jolivet, Verma & Mille, 2007) — Rev. fr. Entomol. 29: 36. (**MNHN**)

99. *Thasyclesgrandis* Gómez-Zurita, 2022 — Zool. Anz. 297: 31. (**MNHW**)

100. *Thasycleslaboulbenei* (Montrouzier, 1861) — Annls. Soc. ent. Fr. 4: 396. (**RBINS**)

101. *Thasyclesmagnus* Gómez-Zurita, 2022 (Fig. 1f) — Zool. Anz. 297: 34. (**MNHW**)

102. *Thasyclespanieensis* (Jolivet, Verma & Mille, 2007) — Rev. fr. Entomol. 29: 79. (**NHM**)

103. *Thasyclespuncticollis* Gómez-Zurita, 2022 — Zool. Anz. 297: 35. (**MNHW**)

104. *Thasyclestenuis* Gómez-Zurita, 2022 — Zool. Anz. 297: 36. (**MNHW**, MNHW, JGZC)

105. *Thasyclesvariegatus* Gómez-Zurita, 2022 — Zool. Anz. 297: 37. (**MNHW**, MNHW, JGZC)

106. *Tricholapitaaphrodita* (Gómez-Zurita, 2014) — Syst. Entomol. 39: 115. (**MNHN**, BPBM, JGZC)

107. *Tricholapitaatlantis* (Platania & Gómez-Zurita, 2020) — Zool. J. Linn. Soc. 189: 15. (**MNHN**)

108. *Tricholapitagaea* (Gómez-Zurita, 2014) — Syst. Entomol. 39: 119. (**MNHN**, BPBM, JGZC, AMS, NRM)

109. *Tricholapitahermes* (Platania & Gómez-Zurita, 2020) — Zool. J. Linn. Soc. 189: 10. (**MNHN**, MNHW)

110. *Tricholapitakronos* (Platania & Gómez-Zurita, 2020) — Zool. J. Linn. Soc. 189: 12. (**MNHN**, MNHW)

111. *Tricholapitamars* (Samuelson, 2010) — Zootaxa 2621: 56. (**BPBM**)

112. *Tricholapitaoceanica* (Platania & Gómez-Zurita, 2020) — Zool. J. Linn. Soc. 189: 23. (**MNHN**, MNHW)

113. *Tricholapitaolympica* (Platania & Gómez-Zurita, 2020) (Fig. 1b) — Zool. J. Linn. Soc. 189: 6. (**MNHN**, MNHW, JGZC)

114. *Tricholapitaouranos* (Platania & Gómez-Zurita, 2020) — Zool. J. Linn. Soc. 189: 24. (**MNHN**, MNHW)

115. *Tricholapitareidi* Gómez-Zurita, Platania & Cardoso, 2020 — Zootaxa 4857: 89. (**MHNW**)

116. *Tricholapitariberai* (Platania & Gómez-Zurita, 2020) — Zool. J. Linn. Soc. 189: 17. (**MNHN**, MNHW)

117. *Tricholapitatridentata* (Platania & Gómez-Zurita, 2020) — Zool. J. Linn. Soc. 189: 4. (**MNHN**, MNHW)

118. Incertae sedis: *Colaspismetallica* Montrouzier, 1861 — Annls. Soc. ent. Fr. 4: 396*.

119. Incertae sedis: *Colaspissolani* Perroud & Montrouzier, 1864 (Fig. 1n) — Annls. Soc. linn. Lyon 11: 208. (**MNHN**)

### ﻿Typophorini

120. *Rhyparidafoaensis* (Jolivet, Verma & Mille, 2007) (Fig. 1l) — Rev. Fr. Entomol. 29: 43. (**MNHN**)

## ﻿Discussion

In this work, we updated the fragmented knowledge on species numbers and taxonomic changes over the past decades on the Eumolpinae of New Caledonia, whereby 120 species in 13 genera should be currently considered, although this figure will be notably increased in the future and many generic attributions changed. This exercise was required, since the Eumolpinae of New Caledonia have seen a rapid increase in the number of taxa proposed recently and in a relatively short amount of time, and also because a relatively important fraction of the global diversity of the subfamily (~ 1.7%) is found in this small archipelago.

The potential magnitude of this diversity was already suggested by Papadopoulou et al (2013), and it is still far from being completely known. Despite the increased rate of species descriptions in the last decade, several clades still need a revision (Papadopoulou et al. 2013) and the archipelago has not been exhaustively explored, which possibly results in a major underestimation of the diversity of New Caledonian Eumolpinae. This is exemplified by the high number of species that are discovered whenever a putative natural group is revised (Gómez-Zurita 2018, 2022; Platania et al. 2020; Platania and Gómez-Zurita 2022; Gómez-Zurita and Pàmies-Harder 2022), and the numbers of species described in the past few years in these revisions is indicative of this trend.

The Eumolpinae of New Caledonia are currently arranged in 13 genera. The assignment to genera is a problem for the group, since notions of diagnostic characters for monophyletic groups only started to be incorporated recently (Gómez-Zurita and Cardoso 2014; Gómez-Zurita 2018; Platania et al. 2020; Gómez-Zurita 2022; Gómez-Zurita and Pàmies-Harder 2022; Platania and Gómez-Zurita 2022). So far, only a handful of genera of New Caledonian Eumolpinae have been assessed based on these principles, including *Acronymolpus*, *Dematotrichus* Gómez-Zurita, 2022, *Kumatoeides* Gómez-Zurita, 2018, *Taophila*, *Thasycles* Chapuis, 1874, and *Tricholapita* Gómez-Zurita & Cardoso, 2020 (Gómez-Zurita and Cardoso 2014; Samuelson 2015; Gómez-Zurita 2018; Platania et al. 2020; Gómez-Zurita 2022; Gómez-Zurita and Pàmies-Harder 2022; Platania and Gómez-Zurita 2022). Most others will require profound reassessment of their boundaries, but not only, since nomenclatural changes are also expected. Some ‘container’ genera, rich in species, usually showing marked differences between them, were proposed based on the general appearance of some species, but their monophyly will be probably challenged when they are studied in greater detail. This would be the case of *Samuelsonia* Jolivet, Verma & Mille, 2007, *Montrouzierella* Jolivet, Verma & Mille, 2007, *Dumbea* Jolivet, Verma & Mille, 2007, and *Dematochroma* Baly, 1864. We have already provided some objective data about the last genus, demonstrating with molecular phylogenetic data and principles that New Caledonian species in this genus must be transferred to other existing or new genera, since they are not monophyletic with the type species of *Dematochroma*, from Lord Howe Island (Gómez-Zurita and Pàmies-Harder 2022). Recent revisions began to address this issue, transferring some of the species to the genera *Dematotrichus* and *Thasycles* (Gómez-Zurita 2022; Gómez-Zurita & Pàmies-Harder 2022). Others, like *Colaspoides* Laporte, 1833, where some current taxa may require synonymization (Jolivet et al. 2013), must be removed from the catalogue, since none of the species of this possibly polyphyletic genus present in the eastern Palaearctic, Oriental, and Neotropical regions, are related to the New Caledonian species, deeply nested within the island radiation (Papadopoulou et al. 2013).

In this work, we also advocate two taxonomic changes that involve species in two of those problematic genera, based on the study of their types. The first one involves the species *Taophilacancellata*, which had been tentatively transferred to *Dematochroma* by Gómez-Zurita and Cardoso (2014), and it can be confirmed as a junior synonym of *Dumbeastriata*. The second illustrates a common problem in previous taxonomic works of New Caledonian Eumolpinae whereby strong sexual dimorphism in some species resulted in the description of males and females as different species or difficulties to recognise males and females as conspecific (Gómez-Zurita 2017a, b). Specifically, *Dematochromapoyensis* is recognised here as the female and *D.theryi* as the male of the same species, and consequently synonymised. Thus, the new synonymies proposed in this work are *Dumbeastriata* Jolivet, Verma & Mille, 2007 = *Taophilacancellata* Samuelson, 2010, syn. nov.; and *Dematochromatheryi* Jolivet, Verma & Mille, 2010 = *Dematochromapoyensis* Jolivet, Verma & Mille, 2010, syn. nov.

The high rate of species descriptions and the expected increase in the number of species, together with expected nomenclatural changes, highlight the importance of this catalogue, which provides data on the current knowledge of Eumolpinae diversity in New Caledonia and the basis for future taxonomic studies, grounded on the study of types, most of them available in just a handful of institutions, as well as phylogenetic information. Thus, the main stimulus of this work is taxonomic in scope, to update and condense in a single place the current taxonomic knowledge on New Caledonian Eumolpinae to aid future biodiversity research in this group. However, species catalogues are also a fundamental tool for conservation biology, since it is obvious that to know what to protect and to design efficient conservation strategies, it is essential to know what species are present in a particular area. This is especially relevant in the case of New Caledonian Eumolpinae, since they represent a highly vulnerable group of New Caledonian biota for several reasons. Beyond the recognised vulnerability of island biotas, among the most threatened in the world, with a third of all terrestrial species at high risk of extinction (Ricketts et al. 2005), all the species and most genera of New Caledonian Eumolpinae are endemic. Moreover, most species studied to date have confined distributions, known from a single locality or group of nearby localities, a condition that can be referred to as micro-endemicity. Indeed, micro-endemicity is a characteristic feature of New Caledonian biodiversity, shared by many different organisms (Caesar et al. 2017) and indicative of their high vulnerability, which together with the extraordinary species richness and the reduced area, led to classify the archipelago as a biodiversity hotspot of high conservation priority (Mittermeier et al. 1999; Myers et al. 2000). Leaf beetles show strong associations with plants with different degrees of ecological specialization. Thus, their vulnerability is also potentially influenced by cascade effects derived from conservation issues of their hosts. The microendemic distributions of many species of both plants and beetles increase exponentially their risk of extinction. Their survival is jeopardized by several factors with global or regional effects, such as climate change (Mora et al. 2013; Wulff et al. 2013; Bellard et al. 2014). However, it is also susceptible to threats resulting from local changes in the environment, which can typically result from human activities, such as mining, timber extraction, or cattle raising, leading to habitat degradation (Pascal et al. 2008; Wulff et al. 2013), but also the introduction of alien species (Gargominy et al. 1996) or other stochastic natural or human-induced events, such as fires (McCoy et al. 1999).

Raising awareness about the high species diversity of Eumolpinae, uncovered thanks to the taxonomic work that is ongoing, building upon the knowledge generated by previous authors, as well as their compromised situation owing to their reduced ranges, would be a first argument to include them in future conservation plans.

## ﻿Additional information

### Author ORCIDs

Leonardo Platania https://orcid.org/0000-0002-1289-5564

Jesús Gómez-Zurita https://orcid.org/0000-0001-7337-6541

### Data availability

All of the data that support the findings of this study are available in the main text or Supplementary Information.
